# Modified creatinine index and risk for long-term infection-related mortality in hemodialysis patients: ten-year outcomes of the Q-Cohort Study

**DOI:** 10.1038/s41598-020-58181-6

**Published:** 2020-01-27

**Authors:** Hokuto Arase, Shunsuke Yamada, Hiroto Hiyamuta, Masatomo Taniguchi, Masanori Tokumoto, Kazuhiko Tsuruya, Toshiaki Nakano, Takanari Kitazono

**Affiliations:** 1 Department of Medicine and Clinical Science, Graduate School of Medical Sciences, Kyushu University, Fukuoka, Japan; 2 Fukuoka Renal Clinic, Fukuoka, Japan; 3 Department of Internal Medicine, Fukuoka Dental College, Fukuoka, Japan; 4 Department of Nephrology, Nara Medical University, Nara, Japan

**Keywords:** End-stage renal disease, Haemodialysis

## Abstract

Modified creatinine (Cr) index, calculated by age, sex, pre-dialysis serum Cr concentration, and Kt/V for urea, is an indicator of skeletal muscle mass in hemodialysis (HD) patients. It remains unknown whether the modified Cr index predicts infection-related mortality in this population. We investigated the association between the modified Cr index and infection-related mortality. A total of 3046 patients registered in the Q-Cohort Study, a multicenter, observational study of HD patients, were analyzed. Associations between sex-specific quartiles (Q1–Q4) of the modified Cr index and the risk for infection-related mortality were analyzed by Cox proportional hazard model. During a median follow-up of 8.8 years, 387 patients died of infection. The estimated risk for infection-related mortality was significantly higher in the lower quartiles (Q1, Q2, and Q3) than in the highest quartile (Q4) as the reference group (hazard ratios and 95% confidence intervals [CI]: Q1, 2.89 [1.70–5.06], Q2, 2.76 [1.72–4.62], and Q3, 1.79 [1.12–2.99]). The hazard ratio (95% CI) for a 1 mg/kg/day decrease in the modified Cr index was 1.18 (1.09–1.27, *P* < 0.01) for infection-related mortality. In conclusion, a lower modified Cr index is associated with an increased risk for long-term infection-related mortality in the HD population.

## Introduction

Hemodialysis (HD) patients have a higher mortality risk than the general population, and infectious disease is a major cause of mortality in patients on maintenance HD^1^. Among the risk factors for infectious disease in this population, malnutrition or protein energy wasting (PEW) is regarded as a critical pathophysiology^2,3^. Malnutrition frequently compromises HD patients and involves decreased skeletal muscle mass and function, termed sarcopenia^2^, eventually leading to increased morbidity and mortality^4–6^. These complex pathologies, involving malnutrition, inflammation, and increased risk for morbidity and mortality, have been integrated into the concept of “malnutrition-inflammation complex syndrome” (MICS)^6^.

In 1995, Canaud *et al*. first proposed the creatinine (Cr) index, which is a convenient indicator of the skeletal muscle mass of HD patients^7^. The original Cr index was derived from Cr kinetic modeling, and these authors have recently proposed a more simplified version of the Cr index requiring only age, sex, pre-dialysis serum Cr concentration, and Kt/V for urea, which is now termed the “modified Cr index”^8^. Medical practitioners can readily and cost-effectively evaluate the skeletal muscle mass of HD patients using this modified Cr index. Furthermore, we recently reported that a lower modified Cr index was significantly associated with higher risks for bone fracture, heart disease, and all-cause mortality from the 4-year follow-up data of the Q-Cohort Study^9,10^. A decrease in skeletal muscle mass reflects the MICS which is considered to be a critical underlying cause of these diverse outcomes. Therefore, we hypothesized that decreased skeletal muscle mass can also predict a risk for infectious disease in HD patients. However, the association between the modified Cr index and infection-related mortality in the HD population remains unclear.

The aim of the present study was to investigate the relationship between estimated skeletal muscle mass, shown by the modified Cr index, and long-term infection-related mortality in HD patients by analyzing the 10-year follow-up data of the Q-Cohort Study. In addition, we determined the predictive value of the modified Cr index using c-statistics, net reclassification improvement (NRI), and integrated discrimination improvement (IDI).

## Methods

### Design of the Q-Cohort study

The Q-Cohort Study was originally designed as a multicenter, longitudinal, observational study for patients with end-stage renal disease undergoing maintenance HD in Japan. The details of this study have been previously described^10–14^. Briefly, 3566 outpatients who were 18 years old or older and underwent regular HD therapy between December 2006 and December 2016 at 39 dialysis facilities were enrolled. All of the patients were scheduled to be followed-up until December 2016. Of the 3566 patients, 520 who had missing data for baseline parameters or outcome were excluded. Therefore, remaining 3046 patients were analyzed in the present study. The present study was performed according to the Ethics of Clinical Research (1975 Declaration of Helsinki). The study protocol was approved by the Kyushu University Hospital Institutional Review Board for Clinical Research (No. 20–31). Written informed consent was provided by all the patients prior to participation in the study. The ethics committee of all participating institutions granted approval to waive the requirement for written informed consent for the additional follow-up surveys from 2011–2016 because of the retrospective nature of this study, which is registered in the University Hospital Medical Information Network (UMIN) clinical trial registry (UMIN ID: 000000556).

### Demographics and biochemical measurements

Demographic and clinical data were ascertained at the baseline. The following data were included; age, sex, presence of diabetic nephropathy, history of cardiovascular events and bone fractures, dialysis history, dialysis time, body mass index (BMI), normalized protein catabolic rate (nPCR), systolic blood pressure, cardiothoracic ratio, and the prescription of erythropoiesis-stimulating agents (ESAs), anti-hypertensives, phosphate-binders, and vitamin D receptor activators (VDRAs). Collection and measurement of samples were performed as previously described^10^. Blood samples were collected by vascular access just prior to initiating dialysis sessions after a 2-day inter-dialytic interval at the baseline and were analyzed to determine hematological and biochemical parameters including hemoglobin and serum concentrations of albumin, total cholesterol, C-reactive protein (CRP), urea nitrogen, Cr, calcium, phosphate, and alkaline phosphatase. These routine parameters were measured using an auto-analyzer with standard procedures at different laboratories, depending on the location of the dialysis centers. Whole or intact PTH assays were used to determine serum parathyroid hormone (PTH) concentrations, and conversion between the two assays was conducted using the Eq. (1)^15^. Payne’s formula (Eq. (2)) was used to calculate corrected serum calcium concentrations^16^. Single-pool Kt/V for urea was used as the index of adequacy of dialysis and was calculated without residual renal function (RRF) in the present study.

### Definition of outcome and covariates

Infection-related mortality was the main outcome of the present study. Definition of infection-related death in the present study was death caused by infectious diseases including respiratory infection (e.g. pneumonia), urinary tract infection (e.g. pyelonephritis), intestinal infection (e.g. enteritis), cardiac infection (e.g. endocarditis), neurologic infection (e.g. meningitis), septicemia, vascular access-related infection, and other infections^17^. The covariate of interest was the modified Cr index^8–10^. Here, the modified Cr index was calculated using the formula (3) based on age, sex, pre-dialysis serum Cr concentrations, and Kt/V for urea^8–10^. We used pre-dialysis serum Cr concentrations that were determined on the first dialysis day of the week. In our previous independent cohort study, a close correlation between the modified Cr index and skeletal muscle mass determined by bioelectrical impedance analysis was validated^9^.

### Statistical analysis

Details of the statistical method used in the present study was also described in our previous study^10^. All continuous parameters are given as the median (interquartile range), while categorical parameters are given as a number (percentage). Because the modified Cr index distributed in different ranges between sexes, the patients were classified into quartiles (Q1–Q4) based on a sex-specific modified Cr index. Trends between quartiles of modified Cr index were examined by the Cochran–Armitage test for categorical parameters and by the Jonckheere–Terpstra test for continuous parameters. Kaplan–Meier survival curves for infection-related mortality among sex-specific modified Cr index quartiles were examined by the log-rank test. Age- and sex-adjusted and multivariable-adjusted hazard ratios (HRs) and 95% confidence intervals (CIs) for infection-related mortality were examined by the Cox proportional hazards model. Proportional hazards were graphically checked by log cumulative hazard plots for infection-related mortality among quartiles of the modified Cr index. Multivariable-adjusted analyses were performed using the following potential confounding factors selected based on *a priori* clinical judgment: age, sex, presence of diabetic nephropathy, dialysis history, nPCR, Kt/V for urea, BMI, and serum concentrations of albumin, urea nitrogen, and CRP. The restricted cubic spline model was used to plot the multivariable-adjusted association between the modified Cr index and HRs with 95% CIs for infection-related mortality. We performed subgroup analysis to estimate the association between a decrease in skeletal muscle mass and infection-related mortality according to subgroups of risk factors. Furthermore, to evaluate the predictive value of the modified Cr index for infection-related mortality, we compared a basic model and a basic model with the modified Cr index by c-statistics calculated using a receiver-operating characteristic curve, NRI, and IDI. A basic model included age, sex, the presence of diabetic nephropathy, dialysis history, nPCR, Kt/V for urea, BMI, and serum concentrations of albumin, urea nitrogen, and CRP as potential risk factors. A difference was considered significant when the *P* value < 0.05. JMP version 13.2 (SAS Institute, Cary, USA) and R software version 3.0.2 (http://cran.rproject.org) were used in statistical analyses.

## Results

### Baseline characteristics

Baseline characteristics among sex-specific quartiles of the modified Cr index were shown in Table 1. Patients with a lower modified Cr index were significantly older, more frequently had diabetic nephropathy and a history of cardiovascular events and bone fractures, and had a shorter dialysis history and dialysis time, lower BMI and nPCR, and higher cardiothoracic ratio than patients with a higher modified Cr index. Patients with a lower modified Cr index showed significantly higher serum CRP and alkaline phosphatase concentrations than patients with a higher modified Cr index. By contrast, patients with a lower modified Cr index showed significantly lower hemoglobin and serum albumin, urea nitrogen, Cr, corrected calcium, phosphate, and PTH concentrations than patients with a higher modified Cr index. Prescription rates of phosphate binders and VDRAs in patients with a lower modified Cr index were significantly lower than in patients with a higher modified Cr index, whereas the prescription rate of ESAs was significantly higher in patients with a lower modified Cr index than in patients with a higher modified Cr index.

**Table 1:** Clinical background at baseline of each group stratified according to sex-specific modified creatinine index quartiles (*n* = 3046).

	Sex-specific quartiles of modified Cr Index, mg/kg/day				
	Q1 (*n* = 759) M: –20.31 F: –17.95	Q2 (*n* = 763) M: 20.32–22.13 F: 17.96–19.43	Q3 (*n* = 762) M: 22.14–24.05 F: 19.44–20.97	Q4 (*n* = 762) M: 24.06– F: 20.98–	*P* for trend
**Demographics and dialysis-related information**					
Modified Cr index (male), mg/kg/day	19.2 (18.5–19.8)	21.3 (20.8–21.7)	23.0 (22.6–23.4)	25.3 (24.6–26.3)	<0.001
Modified Cr index (Female), mg/kg/day	16.9 (16.1–17.4)	18.8 (18.4–19.1)	20.3 (19.8–20.7)	22.1 (21.4–23.0)	<0.001
Age, years	74.9 (68.8–80.9)	68.3 (61.9–74.5)	62.2 (56.6–67.1)	52.6 (44.3–58.7)	<0.001
Female sex	309 (40.7)	309 (40.5)	310 (40.7)	309 (40.6)	0.970
Diabetic nephropathy	311 (41.0)	268 (35.1)	195 (25.6)	103 (13.5)	<0.001
History of cardiovascular events	248 (32.7)	204 (26.7)	151 (19.8)	96 (12.6)	<0.001
History of bone fracture	134 (17.7)	73 (9.6)	56 (7.4)	41 (5.4)	<0.001
Dialysis history, years	2.8 (0.8–6.5)	4.5 (1.9–10.3)	7.1 (3.0–12.7)	8.2 (4.0–14.4)	<0.001
Dialysis time (≥5 hours)	391 (51.5)	473 (62.0)	516 (67.7)	508 (66.7)	<0.001
Kt/V for urea	1.6 (1.4–1.7)	1.6 (1.4–1.8)	1.6 (1.4–1.8)	1.6 (1.4–1.7)	0.545
Body mass index, kg/m^2^	19.8 (18.0–22.0)	20.9 (18.9–23.0)	21.0 (19.0–23.0)	21.6 (19.6–23.8)	<0.001
nPCR, g/kg/day	0.9 (0.8–1.0)	0.9 (0.8–1.0)	1.0 (0.9–1.1)	1.0 (0.9–1.1)	<0.001
Systolic blood pressure, mmHg	153 (138–169)	154 (141–170)	153 (140–168)	151 (136–165)	0.020
Cardiothoracic ratio, %	52.2 (48.1–56.3)	50.6 (47.2–54.1)	50.0 (46.9–53.2)	48.6 (45.9–51.8)	<0.001
**Blood tests**					
Hemoglobin, g/dL	10.3 (9.6–11.1)	10.6 (9.7–11.2)	10.6 (9.9–11.3)	10.7 (10.0–11.4)	<0.001
Serum albumin, g/dL	3.6 (3.3–3.9)	3.8 (3.5–4.0)	3.9 (3.7–4.1)	4.0 (3.8–4.2)	<0.001
Serum total cholesterol, mg/dL	150 (128–175)	152 (132–179)	153 (131–180)	151 (130–176)	0.507
Serum urea nitrogen, mg/dL	57 (46–68)	64 (56–74)	69 (60–78)	73 (65–82)	<0.001
Serum Cr, mg/dL	7.3 (6.4–8.2)	9.5 (8.7–10.4)	11.2 (10.1–12.1)	13.3 (11.9–14.5)	<0.001
Serum C-reactive protein, mg/dL	0.2 (0.1–0.6)	0.1 (0.1–0.4)	0.1 (0.1–0.3)	0.1 (0–0.2)	<0.001
Corrected serum Ca, mg/dL	9.2 (8.8–9.7)	9.3 (8.9–9.8)	9.5 (8.9–10.0)	9.5 (9.0–10.0)	<0.001
Serum phosphate, mg/dL	4.4 (3.7–5.1)	4.7 (4.1–5.5)	5.1 (4.4–5.8)	5.3 (4.6–6.1)	<0.001
Serum alkaline phosphatase, U/L	258 (205–346)	242 (190–313)	226 (177–306)	209 (162–279)	<0.001
Serum PTH (intact assay), pg/mL	85 (40–155)	103 (44–207)	106 (46–215)	129 (57–254)	<0.001
**Medications**					
Prescription of phosphate binders	476 (62.7)	624 (81.8)	677 (88.9)	707 (92.8)	<0.001
Prescription of VDRAs	471 (62.1)	544 (71.3)	571 (74.9)	569 (74.7)	<0.001
Prescription of anti-hypertensives	487 (64.2)	502 (65.8)	493 (64.7)	465 (61.0)	0.176
Prescription of ESAs	684 (90.1)	649 (85.1)	633 (83.1)	594 (78.0)	<0.001

Cr, creatinine; Ca, calcium; ESAs, erythropoiesis-stimulating agents; F, female; M, male; nPCR, normalized protein catabolic rate; PTH, parathyroid hormone; Q, quartile of the modified Cr index; VDRAs, vitamin D receptor activators. Baseline data are expressed as the median (interquartile range) or number (percentage). The Cochran–Armitage test was used to determine P for a trend of categorical variables. The Jonckheere–Terpstra test was used to determine P for a trend of continuous variables. A two-tailed P value < 0.05 was considered statistically significant.

### Association between the modified creatinine index and the nutritional marker

**Figure 1:**
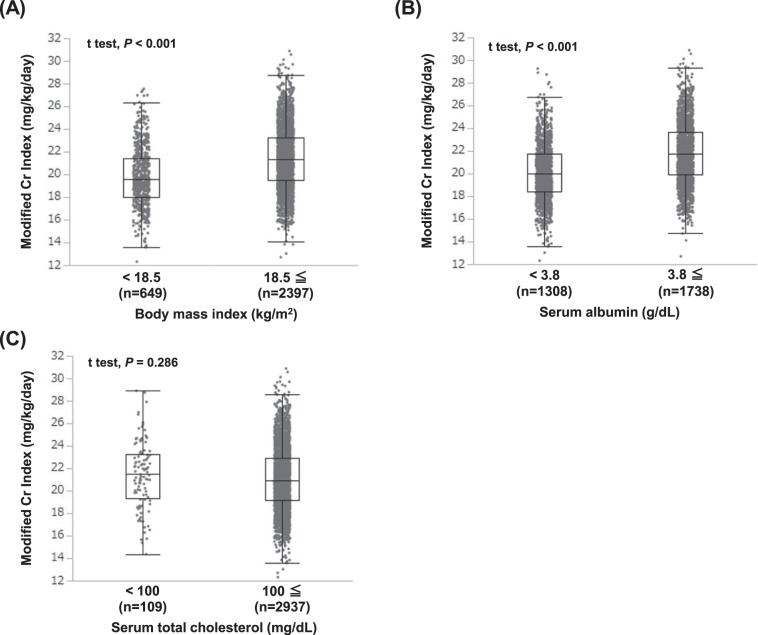
Distribution of the modified Cr index in each group stratified by nutritional markers including (**A**) body mass index, (**B**) serum albumin concentration, and (**C**) serum total cholesterol concentration. Unpaired t-test was used to analyze statistical differences. A two-tailed *P* value < 0.05 was considered statistically significant. Abbreviations: Cr, creatinine.

Because lower serum albumin and total cholesterol concentrations, lower BMI, and decreased skeletal muscle mass are included in the criteria of PEW^18,19^, the association between modified Cr index and nutritional markers was examined. The mean modified Cr index was significantly lower in both the lower serum albumin concentration and lower BMI groups compared with the respective higher ones (both *P* < 0.01; t test, Fig. 1), while the mean modified Cr index was not different between the higher and lower serum total cholesterol concentration groups.

### Association between the modified creatinine index and the risk for infection-related mortality

**Figure 2:**
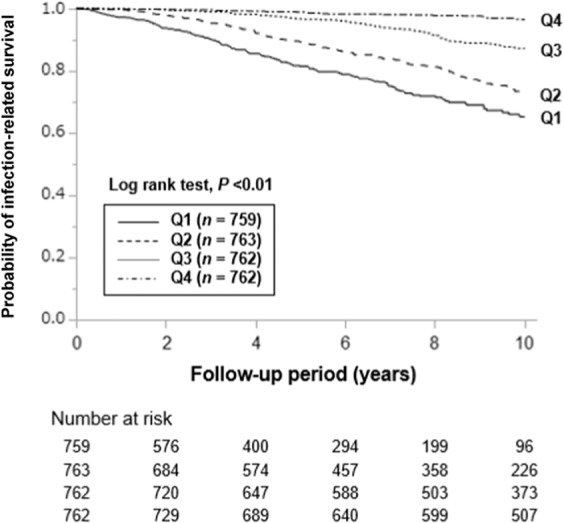
Kaplan-Meier curves for the incidence of infection-related mortality in each group stratified by the sex-specific modified Cr index. Patients were stratified into sex-specific quartiles according to the modified Cr index. Log-rank test was used to analyze statistical differences. A two-tailed *P* value < 0.05 was considered statistically significant. Abbreviations: Cr, creatinine; Q, quartile of the modified Cr index.

**Figure 3:**
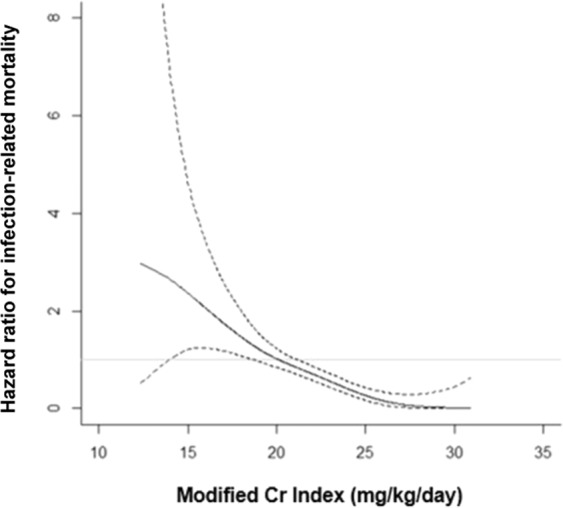
Multivariable-adjusted restricted cubic spline plots of the hazard ratio (HR) for infection-related mortality according to the modified Cr index. The solid line represents the HR and the dotted lines the 95% confidence intervals. The multivariable-adjusted model was adjusted for age, sex, presence of diabetic nephropathy, dialysis history, normalized protein catabolic rate, Kt/V for urea, body mass index, and serum concentrations of albumin, urea nitrogen, and C-reactive protein. Abbreviations: Cr, creatinine.

During a median observational period of 8.8 years (interquartile range: 4.1–10.0 years), 387 patients died of infection and 1503 patients of any cause. The incidence of infection-related mortality was highest in the lowest modified Cr index quartile (Q1) (Table 2). Kaplan–Meier curves showed significantly higher incidence rates of infection-related mortality at lower modified Cr index quartiles than at higher modified Cr index quartiles (*P* < 0.01; log-rank test, Fig. 2). In the age- and sex-adjusted and multivariable-adjusted Cox proportional hazards models, patients with lower modified Cr index quartiles (Q1, Q2, and Q3) displayed higher adjusted HRs for the incidence rates of infection-related mortality compared with the reference group (Q4) (Table 3). The HR (95% CI) for a 1 mg/kg/day decrease in the modified Cr index was 1.18 (1.09–1.27, *P* < 0.01) for infection-related mortality. The continuous multivariable-adjusted association between the modified Cr index and infection-related mortality was also shown by a restricted cubic spline curve. The HRs for infection-related mortality increased incrementally with a decrease in the modified Cr index (Fig. 3).

**Table 2:** Outcomes during the observation period in each group stratified according to sex-specific modified creatinine index quartiles (*n* = 3046).

	Sex-specific quartiles of modified Cr index, mg/kg/day				
	Q1 (*n* = 759) M: –20.31 F: –17.95	Q2 (*n* = 763) M: 20.32–22.13 F: 17.96–19.43	Q3 (*n* = 762) M: 22.14–24.05 F: 19.44–20.97	Q4 (*n* = 762) M: 24.06– F: 20.98–	*P* for trend
Infection-related death	149 (19.6)	142 (18.6)	73 (9.6)	23 (3.0)	<0.001
All-cause death	594 (78.3)	471 (61.7)	305 (40.0)	133 (17.5)	<0.001

Cr, creatinine; F, female; M, male; Q, quartile of the modified Cr index. Outcomes during the observation period are expressed as number (percentage). The Cochran–Armitage test was used to determine P for the trend of outcomes. A two-tailed P value < 0.05 was considered statistically significant.

**Table 3:** Hazard ratios for Infection-related mortality in each group stratified by sex-specific modified creatinine index quartiles (*n* = 3046).

	Age- and sex-adjusted			Multivariable-adjusted		
	HR (95% CI)	*P* -value	*P* for trend	HR (95% CI)	*P*-value	*P* for trend
Infection-related mortality			<0.001			<0.001
Q1	4.31 (2.66–7.26)	<0.001		2.89 (1.70–5.06)	<0.001	
Q2	3.59 (5.59–5.93)	<0.001		2.76 (1.72–4.62)	<0.001	
Q3	2.09 (2.25–3.48)	0.002		1.79 (1.12–2.99)	0.014	
Q4	1.00 (reference)	—		1.00 (reference)	—	
Every 1-mg/kg/day decrease in the modified Cr index	1.23 (1.17–1.31)	<0.001		1.18 (1.09–1.27)	<0.001	

CI, confidence interval; Cr, creatinine; nPCR, normalized protein catabolic rate, HR, hazard ratio; Q, quartile of the modified creatinine index. Age- and sex-adjusted and multivariable-adjusted HRs were analyzed by Cox proportional hazards risk model. The covariates for assessing infection-related mortality included age, sex, the presence of diabetic nephropathy, dialysis history, nPCR, Kt/V for urea, body mass index, and serum concentrations of albumin, urea nitrogen, and C-reactive protein. A two-tailed *P* value < 0.05 was considered statistically significant.

### Subgroup analysis

To evaluate the heterogeneity of the association between the modified Cr index and baseline parameters regarding infection-related mortality, subgroup analyses were performed. The association between a lower modified Cr index and infection-related mortality was reduced in patients with a shorter dialysis history compared with patients with a longer dialysis history (*P* for interaction <0.001) (Fig. S1).

### Predictive value of the modified creatinine index

To evaluate the predictive value of the modified Cr index, we compared c-statistics, NRI, and IDI for infection-related mortality between the basic model with and without the modified Cr index. Although c-statistics and IDI for infection-related mortality did not significantly increase by the addition of the modified Cr index to the basic model, the NRI for infection-related mortality showed a slightly but significantly better performance in the basic model with the modified Cr index than without it (Fig. S2 and Table S1).

## Discussion

In the present cohort study of patients on maintenance HD, we demonstrated that the modified Cr index was significantly associated with nutritional markers including serum albumin concentration and BMI, both of which are included in the PEW criteria. Furthermore, we demonstrated that a lower modified Cr index was significantly associated with a higher 10-year infection-related mortality, even after rigorous adjustment for potential confounding factors. Our study suggests that the modified Cr index, an indicator of skeletal muscle mass, can be used as a nutritional marker of HD patients, which predicts long-term prognosis of infectious mortality in this population.

Skeletal muscle is an essential organ that has multiple functions, not limited to producing mechanical movement or in skeletal support of the body. Demonstrating the importance of such multiple functions of skeletal muscle, accumulating evidence has revealed that sarcopenia is a strong risk factor for adverse events in HD patients^5,20^. One of the reasonable explanations for the association between a lower modified Cr index and infection-related mortality is the multifaceted function of skeletal muscle on immunity. First, skeletal muscle is the most important organ that induces thermogenesis^21^. When patients contract an infectious disease, they strengthen immunocompetence by raising body temperature mainly with frequent muscle contraction during shivering^22^. Hyperthermia inhibits viral and bacterial proliferation, while promoting leukocyte and macrophage migration and function. Skeletal muscle also plays an important role in energy storage^23^. In an infectious state, various types of hormones and cytokines are induced by stress and lead to the hypercatabolism of skeletal muscle and adipose tissue to manage a latent starved condition. Conversely, decreased skeletal muscle mass results in insufficient thermogenesis and energy expenditure, which is a potential cause of increased severity of infectious disease.

Another possible explanation for our results is the role of exercise-induced, skeletal muscle-derived cytokines termed myokines^24^. Recently, evidence from both clinical and basic research have shown protective effects of myokines in cardiovascular disease and mortality^25,26^. Furthermore, several studies demonstrated possible direct effects of myokines on immunity^27^. Although a detailed mechanism has not been clarified, the protective effects of myokines on various organs, including the immune system, should be noted.

Furthermore, we need to consider the underlying mechanism regarding the association between the modified Cr index and our clinical outcome other than through direct effects of skeletal muscle. In our present and previous studies, we demonstrated that the modified Cr index strongly correlates with nutritional and inflammatory markers, including the BMI, nPCR, and serum albumin and CRP concentrations^10^. That is, these associations show that a decrease in skeletal muscle mass evidenced by a lower modified Cr index partly reflects the MICS. Many previous reports demonstrated that MICS is a strong predictor for a worse prognosis of all-cause mortality, cardiovascular events, and infectious disease in HD patients^6,28,29^.Therefore, our results suggest that the modified Cr index serves as a comprehensive indicator of MICS and of skeletal muscle mass, and has a good correlation with diverse clinically important outcomes, including infectious mortality.

Subgroup analyses demonstrated that a shorter dialysis history reduced the association between sarcopenia and infection-related mortality. One possible explanation for this heterogeneity may be that RRF was not included when calculating the modified Cr index. Because the serum Cr concentration is determined by RRF and dialysis adequacy in HD patients^30^, the skeletal muscle mass of patients with a shorter dialysis history, who are likely to have a higher RRF, may not be evaluated accurately by the modified Cr index. Furthermore, because medium-sized proteins, including cytokines, are difficult to eliminate in HD, renal excretion of cytokines is also important in HD patients^31,32^. Therefore, the ability to eliminate inflammatory cytokines from the body may be greater in patients with a higher RRF than those with a lower RRF, leading to a lesser impact of MICS on infection-related mortality in patients with a higher RRF.

Because our study has a large number of subjects with wide-ranging inclusion criteria, our data should better reflect the risk for infection-related mortality in a real-world HD population. No large-scale cohort study has ever shown an association between the modified Cr index and infection-related mortality in HD patients. Furthermore, we previously demonstrated the relationships between the modified Cr index and various outcomes, including bone fractures, heart disease, and all-cause mortality^9,10^. Although data from France was initially used to create the modified Cr index^8^, our results displayed the universal usefulness of the modified Cr index independent of the patients’ racial group.

Our study had several limitations. First, the characteristics, including the modified Cr index, were measured only once at baseline. Therefore, misclassification bias cannot be totally excluded. Furthermore, because the modified Cr index might have changed during the observation period, a time-dependent model is better for survival analysis and is required in any future studies. Second, we did not classify the type of infection, whether pneumonia, urinary tract infection, or vascular access-related infection. It is possible that the modified Cr index may impact differently on the different causes of infection-related mortality. Third, as previously described, RRF was not measured in the present study. RRF might affect serum Cr concentration and Kt/V for urea, both of which were included in the formula of the modified Cr index. Therefore, the modified Cr index might not reflect skeletal muscle mass accurately particularly in patients with preserved RRF. Fourth, a causal relationship between the modified Cr index and outcome could not be concluded because the study was designed as an observational study. Finally, although we adjusted for potential confounders, known and unknown residual confounders might modify our results. Despite these limitations, we believe that our results provide useful information on the association between the modified Cr index, skeletal muscle mass indicator, and critical clinical outcome in patients on maintenance HD.

In conclusion, our findings demonstrated that decreased skeletal muscle mass, represented by a lower modified Cr index, was significantly associated with a greater long-term risk for infection-related mortality in HD patients. Furthermore, the modified Cr index was a good predictor for these outcomes in the HD population. Future research is needed to answer the question of whether intervention to increase skeletal muscle mass is beneficial to decrease diverse life-threatening outcomes in HD patients.

### Equations

1 $${\rm{intact}}\,{\rm{PTH}}({\rm{pg}}/{\rm{mL}})=1.7\times {\rm{whole}}\,{\rm{PTH}}({\rm{pg}}/{\rm{mL}})$$

2 $${\rm{corrected}}\,{\rm{serum}}\,{\rm{calcium}}={\rm{serum}}\,{\rm{calcium}}\,{\rm{concentration}}+(4-{\rm{serum}}\,{\rm{albumin}}\,{\rm{concentration}})$$

3 $$\begin{array}{c}{\rm{modified}}\,{\rm{Cr}}\,{\rm{index}}\,({\rm{mg}}/{\rm{kg}}/{\rm{day}})=16.21+(1.12\times [1\,{\rm{if}}\,{\rm{male}};\,0\,{\rm{if}}\,{\rm{female}}])\\ -(0.06\times {\rm{age}}({\rm{years}}))-(0.08\times {\rm{single}} \mbox{-} {\rm{pooled}}\,{\rm{Kt}}/{\rm{V}}\,{\rm{for}}\,{\rm{urea}})\\ +\,(0.009\times {\rm{serum}}\,{\rm{Cr}}\,{\rm{concentration}}\,{\rm{before}}\,{\rm{dialysis}}({\rm{\mu }}{\rm{mol}}/{\rm{L}}))\end{array}$$

## Supplementary information

Supplementary Information.

## Data Availability

The datasets generated during and/or analyzed during the current study are available from the corresponding author on reasonable request.
