# Supplementary material for: Modified creatinine index and risk for long-term infection-related mortality in hemodialysis patients: ten-year outcomes of the Q-Cohort Study
Source: Sci Rep. 2020 Jan 27;10:1241. doi: 10.1038/s41598-020-58181-6 (PMC6985259; doi:10.1038/s41598-020-58181-6)
Supplement: Supplementary file 1 — Supplementary Information. [file 41598_2020_58181_MOESM1_ESM.docx]

**ONLINE SUPPLEMENT**

**Modified creatinine index and risk for long-term infection-related mortality in hemodialysis patients: ten-year outcomes of the Q-Cohort Study**

Hokuto Arase ^1^, Shunsuke Yamada ^1^, Hiroto Hiyamuta ^1^, Masatomo Taniguchi ^2^, Masanori Tokumoto ^3^, Kazuhiko Tsuruya ^4^, Toshiaki Nakano ^1^*****, and Takanari Kitazono ^1^

1 Department of Medicine and Clinical Science, Graduate School of Medical Sciences, Kyushu University, Fukuoka, Japan

2 Fukuoka Renal Clinic, Fukuoka, Japan

3 Department of Internal Medicine, Fukuoka Dental College, Fukuoka, Japan

4 Department of Nephrology, Nara Medical University, Nara, Japan

***Corresponding author**

Toshiaki Nakano, MD, PhD.

Department of Medicine and Clinical Science, Graduate School of Medical Sciences, Kyushu University, 3-1-1 Maidashi, Higashi-ku, Fukuoka 812-8582, Japan.

Tel.: +81-92-642-5843; Fax: +81-92-642-5846

E-mail: toshink@med.kyushu-u.ac.jp


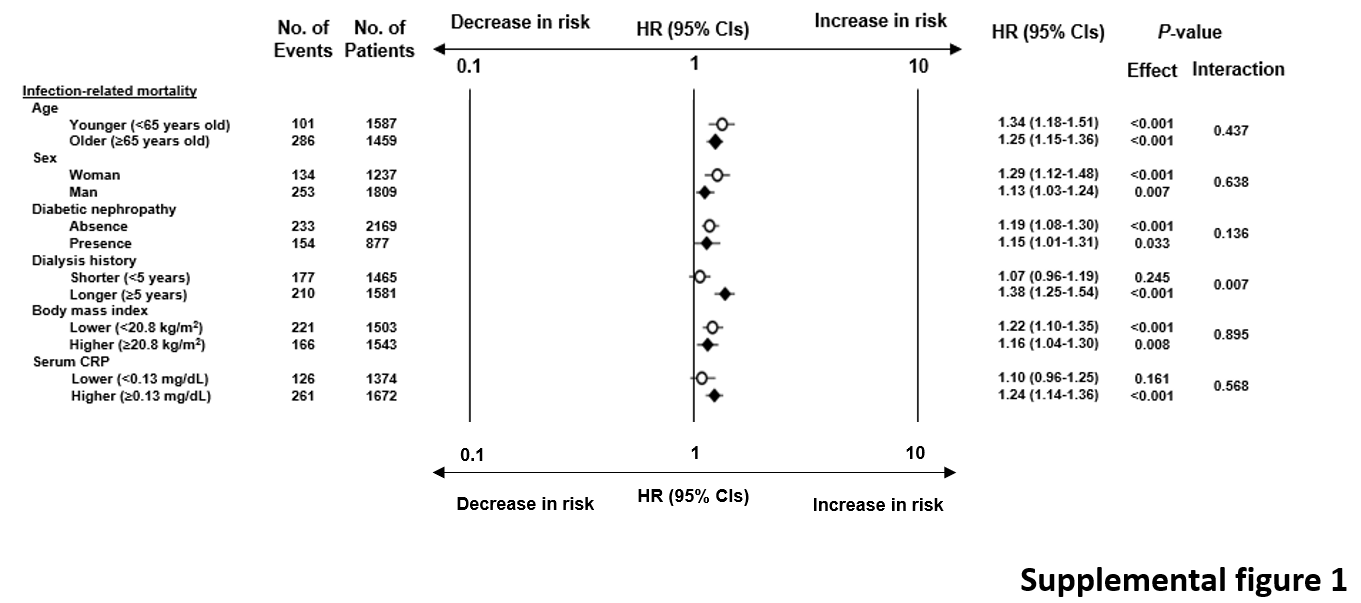


**Supplemental Figure 1.** Multivariable-adjusted hazard ratio (HR) and 95% confidence intervals (CIs) for infection-related mortality by a decrease of 1 mg/kg/day of the modified Cr index in subgroups stratified by baseline characteristics. Open circles and filled rhombi denote point estimate of HRs, and error bars represent 95% CIs. Variables relevant to subgroups were excluded from each model. A two-tailed *P* value < 0.05 was considered statistically significant. Abbreviations: Cr, creatinine; CRP, C-reactive protein.


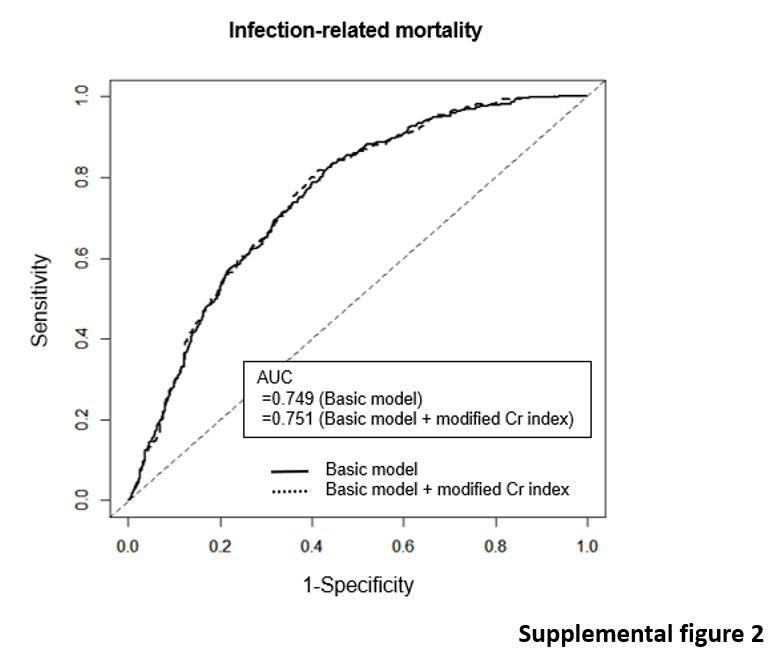


**Supplemental Figure 2.** Comparison of the accuracy of the risk assessment for infection-related mortality between the basic model without or with the modified Cr index. The basic model for infection-related mortality included age, sex, the presence of diabetic nephropathy, dialysis history, nPCR, Kt/V for urea, body mass index, and serum concentrations of albumin, urea nitrogen, and C-reactive protein. Abbreviations: AUC, area under the curve; Cr, creatinine; nPCR, normalized protein catabolic rate.

**Supplemental Table 1.** Predictive value of each model for infection-related mortality using c-statistics, net reclassification improvement, and integrated discrimination improvement (*n* = 3046)

| Predictive models | c-statistic | *P* value | NRI | *P* value | IDI | *P* value |
| --- | --- | --- | --- | --- | --- | --- |
| Infection-related mortality |  |  |  |  |  |  |
| Basic model | 0.749 (0.726–0.773) | – | – | – | – | – |
| Basic model + modified Cr index | 0.751 (0.728–0.775) | 0.320 | 0.111 | 0.041 | 0.001 | 0.20 |

Cr, creatinine; nPCR, normalized protein catabolic rate; NRI, net reclassification improvement; IDI, integrated discrimination improvement.

The basic model for infection-related mortality included age, sex, the presence of diabetic nephropathy, dialysis history, nPCR, Kt/V for urea, body mass index, and serum concentrations of albumin, urea nitrogen, and C-reactive protein. A two-tailed *P* value < 0.05 was considered statistically significant.
